# NHS health checks: a cross- sectional observational study on equity of uptake and outcomes

**DOI:** 10.1186/s12913-018-3027-8

**Published:** 2018-04-03

**Authors:** N. Coghill, L. Garside, A. A. Montgomery, G. Feder, J. Horwood

**Affiliations:** 1 Department for Health, University of Bath, Claverton Down, Bath, BA2 7AY UK; 2 Population Health Sciences, University of Bristol, Whatley Road, Bristol, BS8 2PS UK; 3 School of Medicine, University of Nottingham, Nottingham, NG7 2UH UK; 4 National Institute for Health Research, Collaborations for Leadership in Applied Health Research and Care West (NIHR CLAHRC West), University Hospitals Bristol NHS Foundation Trust, Whitefriars, Lewins Mead, Bristol, BS1 2NT UK

**Keywords:** NHS health checks, Primary care, Inequalities, Public health, Gender, Ethnicity

## Abstract

**Background:**

The National Health Checks programme aims to reduce the incidence of cardiovascular diseases and health inequalities in England. We assessed equity of uptake and outcomes from NHS Health Checks in general practices in Bristol, UK.

**Methods:**

A cross-sectional study using patient-level data, from 38 general practices. We descriptively analysed the socioeconomic status (SES) of patients invited and the SES and ethnicity of those attending. Logistic regression was used to test associations between invitation and attendance, with population characteristics.

**Results:**

Between June 2010 to October 2014, 31,881 patients were invited, and 13,733 NHS Health Checks completed. 47% of patients invited from the three least and 39% from the two most-deprived index of multiple deprivation quintiles, completed a Check. Proportions of invited patients, by ethnicity were 64% non-black and Asian and 31% black and Asian. Men were less likely to attend than women (OR 0.73, 95% confidence interval 0.67 to 0.80), as were patients ≤ 49 compared to ≥ 70 years (OR 0.40, 95% confidence interval 0.65 to 0.83).

After controlling for SES and population characteristics, compared to patients with low CVD risk, high risk patients were more likely to be prescribed cardiovascular drugs (OR 6.2, 95% confidence interval 4.51 to 8.40). Compared to men, women (OR 01.18, 95% confidence interval 1.03 to 1.35) were more likely to be prescribed cardiovascular drugs, as were those ≤ 49 years (50–59 years, OR 1.42, 95% confidence intervals 1.13–1.79, 60–69 years, OR 1.60, 95% confidence intervals, 1.22–2.10, ≥ 70 years, OR 1.64, 95% confidence intervals, 1.14 to 2.35).

Controlling for population characteristics, the following groups were most likely to be referred to lifestyle services: younger women (OR 2.22, 95% CI 1.69 to 2.94), those in the most deprived IMD quintile (OR 3.22, 95% CI 1.63 to 6.36) and those at highest risk of CVD (OR, 2.77, 95% CI 1.91 to 4.02).

**Conclusions:**

We found no statistically significant evidence of inequity in attendance for an NHS Health Check by SES. Being older or a woman were associated with better attendance. Targeting men, younger patients and ethnic minority groups may improve equity in uptake for NHS Health Checks.

## Background

Cardiovascular Disease (CVD) is one of the leading causes of premature mortality and morbidity in the UK [1]. The estimated annual cost of CVD in the UK is £15 billion, but likely to rise to >£18 billion per year by 2020 [2]. Smoking, being overweight, defined as having a BMI ≥ 25, drinking more than 14 units of alcohol a week on a regular basis, and being physically inactive, defined as doing less than 150 min of moderate intensity physical activity per week are considered risk factors for CVD [3].

The NHS Health Checks programme [4], introduced in 2009, is a national initiative, targeting patients aged 40–75, who are not on a disease register for CVD, diabetes or chronic kidney disease. The programme is aimed at the early prevention of type two diabetes, stroke, heart disease and chronic kidney disease, using a combination of risk assessment, communication of risk and risk management. Practices are advised to invite 20% of their eligible population per year for an NHS Health Check, with the aim of inviting their entire eligible practice population, over a five-year period. According to Public Health England, the UK government body who oversee the delivery of this programme, their economic modelling showed a requirement for 75% uptake to provide optimal economic effects on the NHS budget.

The information obtained from the NHS Health Check, including ethnicity, and level of deprivation, measured using the Townsend score, is used to calculate and present attendees with a QRisk score. This score indicates their likely risk of having a heart attack or stroke over the subsequent 10 years.

In line with the NHS Health Checks practice guidelines, patients identified with a QRisk > 10% are offered behavioural advice and interventions, on the day of their check. These include verbal lifestyle advice and a referral to local behavioural lifestyle modification services, for example smoking cessation clinics, weight management and physical activity programmes. Patients whose tests suggest chronic conditions such as chronic kidney disease or hypertension are referred to their GP for formal diagnosis and treatment. Moreover, it is encouraged that eligible patients are placed on the relevant disease register to promote continued monitoring, by the GP practice, of any newly diagnosed condition related to CVD, in-line with government directives.

The incidence of CVD is associated with health inequalities [5, 6]. In the UK, mortality from CVD is 50% higher in those who are in the most deprived fifth of the population compared to those in the most affluent fifth of the population [6]. Some ethnic minority groups are at greater risk of CVD, with mortality rates almost 50% higher in south Asian compared to non-south Asian populations [7]. The NHS Health Checks programme aims to contribute towards reducing health inequalities in England [8, 9] through targeted approaches to encourage uptake in low income and ethnic minority groups [10].

There is some evidence that uptake of NHS Health Checks has been similar across all socioeconomic groups [11, 12]. However, uptake in certain ethnic minority groups has been poor [11].

This evaluation assessed the association between invitation and uptake of NHS Health Checks, and age, sex, ethnicity and level of deprivation, using routinely collected data from general practices in Bristol, a city in the southwest of England, where 16% of the total population are from Black, Asian and other minority ethnic groups [13].

## Methods

This was a cross-sectional, observational study of eligible patients registered at general practices in Bristol, from 18th February 2010 to 23rd October 2014. Eligible patients included those aged 40–74 without pre-existing CVD, hypertension, ischaemic heart disease, stroke or transient ischaemic attacks, atrial fibrillation, heart failure, peripheral arterial disease, chronic kidney disease (CKD), familial hypercholesterolaemia, diabetes and current statin prescription.

Thirty eight of the 52 general practices in Bristol gave consent for their electronic patient record database to be interrogated remotely by the local Commissioning Support Unit (CSU), to enable data extraction. The CSU is an independent organisation, commissioned by Public Health Bristol to provide selected data to local health care commissioners.

We obtained anonymised individual level data from participating practices. Identifiable data such as date of birth was converted to age and deprivation status was converted from postcode to lower super output area (LSOA), a small geographical area with a mean population size of 1500 people. This was further converted to a national index of multiple deprivation (IMD) [14] at source and grouped into quintiles based on national distribution. We intended to ascribe ethnicity based on the 2001 census definitions [15] from data recorded in patients electronic medical records. We obtained data on cardiovascular medication prescribed up to three months following completion of the NHS Health Check. Medication was categorised into: thiazides and related diuretics, beta blockers, other anti-hypertensives and heart failure drugs, antiplatelet drugs, antifibrinolytic and haemostatic drugs and lipid regulating drugs. In addition, we received data on referrals to lifestyle services up to eight weeks following completion of the NHS Health Check. Ten-year risk of cardiovascular disease was estimated using the QRISK tool [16]. This score is calculated from patient reported family history, age, gender, ethnicity, socio-economic status, and selected physiological measurements, and can be categorised as < 10% (low), 10%-20 (medium) or > 20% (high).

All eligible patients who received an invitation for an NHS Health Check and were between the ages 39 and 75 years were included in the analysis. Although the age criteria to receive an NHS Health Check is 40–74, the age range used in the analysis was extended to 39–75 years. This ensured that the lower age captured patients who aged 39 at the time of being invited for an NHS Health Check, were aged 40 by the time the NHS Health Check was completed. The upper age enabled us to capture patients who were aged 74 at the time of being invited for an NHS Health Check but were aged 75 by the time they completed their NHS Health Check.

Age was divided into five age-groups as described in Table 1, IMD was divided in to five quintiles, each representing 20% of the population of England. Higher deprivation scores and quintiles represented higher levels of deprivation. Ethnicity was categorised in line with Good Clinical Practice Guidelines  [17], which are based on the 2001 national census categories [15].

**Table 1:** Characteristics of patients invited for an NHS Health Check (June 2010-October 2014)

Invited for an NHS Health Check	
Gender, *n* (%)	
* N*	31,881
Female	15,316 (48)
Male	16,565 (52)
Age in years ^a^	
* N*	31,764
Mean (SD)	52.4 (9.8)
Median (25th,75th)	50 (44, 60)
Min, max	39,75
≤ 49 years *n* (%)	13,920 (43.7)
50–59 years *n* (%)	9443 (29.6)
60–69 years *n* (%)	6167 (19.3)
70–79 years *n* (%)	2234 (7.0)
Missing *n* (%)	117 (0.4)
Ethnicity ^b^, *n* (%)	
* N*	31,881
White: British	18,774 (58.9)
White: Irish	224 (0.7)
White: Other White	1346 (4.2)
Mixed: White & Black Caribbean	194 (0.6)
Mixed: White & Black African	90 (0.3)
Mixed: White & Asian	101 (0.3)
Mixed: Other Mixed	153 (0.5)
Asian/Asian British: Indian	365 (1.1)
Asian/Asian British: Pakistani	364 (1.1)
Asian/Asian British: Bangladeshi	83 (0.3)
Asian/Asian British: Other Asian	154 (0.5)
Other Black: Caribbean	358 (1.1)
Other Black: African	532 (1.7)
Other Black: Other	244 (0.8)
Other: Chinese	157 (0.5)
Other: Any Other Ethnic Group	216 (0.7)
Not Stated or Missing	8526 (26.7)
National Index of Multiple Deprivation	
* N*	31,824
Mean (SD)	24.8,15.7
Median (25th, 75th)	23 (12, 33)
Min, max	2, 70
National IMD quintile, *n* (%)	
Quintile 1 (least deprived)	4029 (12.6)
Quintile 2	6134 (19.2)
Quintile 3	4847 (15.2)
Quintile 4	9148 (28.7)
Quintile 5 (most deprived)	7666 (24.0)
Missing	57 (0.2)

Notes

^a^ Age in years at NHS health check or at first invite

^b^ In subsequent analyses, ethnicity is further aggregated from 16 into 5 categories. These are as indicated in the first part of the ethnicity label: White; Mixed; Asian/Asian British; Other Black; Other. This is in addition to the category ‘Not stated or missing’

Stata V13.1 was used to analyse all data. We used appropriate descriptive statistics to summarise participant characteristics. Logistic regression models were used to investigate associations between participant characteristics and invitation or attendance for an NHS Health Check, and with management of cardiovascular risk factors including prescribed medication and referral to behavioural lifestyle services.

Reporting of this study conforms to STROBE recommendations for observational studies [18].

## Results

On 1st February 2010, out of the fifty-two general practices in Bristol, 110,288 patients were eligible for an NHS Health Check. Thirty-eight practices agreed to provide data and 14 declined. IMD scores were normally distributed across both the practices included and those not included in this evaluation.

### Invitations

A total of 31,881 patients were invited for an NHS Health Check during the data collection period, June 2010–October 2014 (Table 1). Some practices prioritised high-risk patients and other practices did not stratify their invited patients based on risk or age, however, we were unable to quantify this aspect by practice.

### Attendance

13,733 NHS Health Checks were completed on patients over the data collection period (Table 2), representing 43.2% of patients invited. Over the data-collection period the number of NHS Health Checks completed across the 38 practices increased from 68.4 to 459.8 NHS Health  Checks per month.

**Table 2:** Characteristics of Patients who attended for an NHS Health Check (June 2010–October 2014)

Attended for an NHS Health Check	No	Yes	Crude OR ^a^	Adjusted OR ^b^	95% CI ^c^		*p*-value ^d^
Age ^e^, *n* (%)							
*N*	***18,031***	***13,733***	***31,764***				
≤ 49 years	9013 (50.0)	4907 (35.7)					< 0.001
50–59 years	5379 (29.8)	4064 (29.6)	1.39	1.36	1.21	1.53	
60–69 years	2731 (15.1)	3436 (25.0)	2.31	2.19	1.8	2.68	
≥ 70 years	908 (5.0)	1326 (9.7)	2.68	2.53	1.89	3.39	
Gender, n (%)							
*N*	***18,148***	***13,733***	***31,881***				
Female	8043 (44.3)	7273 (53.0)					< 0.001
Male	10,105 (55.7)	6460 (47.0)	0.71	0.73	0.67	0.8	
National Index of Multiple Deprivation, n (%)							
*N*	***18,120***	***13,704***	***31,824***				
Quintile 1 (least deprived)	2124 (11.7)	1905 (13.9)					0.053
Quintile 2	3069 (16.9)	3065 (22.4)	1.11	1.15	0.87	1.53	
Quintile 3	2712 (15.0)	2135 (15.6)	0.88	0.94	0.66	1.33	
Quintile 4	5504 (30.4)	3644 (26.6)	0.74	0.81	0.52	1.26	
Quintile 5 (most deprived)	4711 (26.0)	2955 (21.6)	0.70	0.8	0.52	1.24	
Ethnicity, *n* (%)							
*N*	***18,148***	***13,733***					
White	8732 (48.1)	11,612 (84.6)					
Mixed	155 (0.9)	383 (2.8)					
Asian or British Asian	442 (2.4)	524 (3.8)					
Other Black	620 (3.4)	514 (3.7)					
Missing	8199 (45.2)	700 (5.1)					

Notes

a. Odds ratios reported from logistic regressions clustered by practice (38 clusters)

b. Adjusted odds ratios reported from logistic regression clustered by practice (38 clusters) for model with age, gender and IMD. Ethnicity is not stated or missing for a large proportion of patients not attending an NHS Health Check (44.5%), hence not included in the model

c. 95% confidence intervals reported for adjusted odds ratios (model with age, gender and IMD)

d. *p*-values reported for Wald tests in model with age, gender and IMD

e. The NHS Health Check program is not intended for under 40s, however a small number of patients were reported as < 40

Bold data represent absolute totals.

More women compared to men, older compared to younger patients and higher compared to lower socio-economic status patients attended for their NHS Health Checks (Table 2).

Due to the poor recording of ethnicity by practices, particularly in those patients who did not attend for their NHS Health Check, it was not possible to estimate the association between ethnicity and attendance. However, out of those who did attend, 10.3% of attenders were from Black and Asian minority ethnic groups (BAME).

Over 45% of patients invited for a Health Check and who did not attend, did not have their ethnicity recorded, compared with 5% of attenders (Table 2), precluding calculation of the odds ratios for ethnicity in attenders versus non-attenders.

There was no statistically significant difference in attendance or not, by IMD quintile. However, there was a non-statistically significant trend for patients in the most deprived IMD quintiles, to be less likely to attend for their NHS Health Check compared to patients from less deprived IMD quintiles (*P* = 0.053); but, 95% confidence intervals crossed one, for all reported IMD quintiles. (Table 2).

The distribution of QRisk scores across IMD quintiles was similar. The majority of attendees (61.6%) had a low QRisk score, followed by 25.0% with a medium and 12.5% with a high QRisk.

### Management of cardiovascular risk

*Prescription of cardiovascular medications*: 1161 new prescriptions for cardiovascular medications were issued within three months following completion of an NHS Health Check. Increasing age, being female and having a high or medium QRisk score compared to a low Qrisk score, were associated with these prescriptions (Table 3).

**Table 3:** Characteristics of patients prescribed (Yes) not prescribed (No) cardiovascular drug*

Prescribed any CVD drug within three months following NHS Health Checks	Maximum N (*n* = 13,733)			Complete cases (*n* = 12,606)						
	No	Yes	Crude OR ^a^	No	Yes	Crude OR ^a^	Adjusted OR ^b^	95% CI ^c^		p-value ^d^
Age ^e^, *n* (%)										
*N*	***12,572***	***1161***	***13,733***	***11,565***	***1041***	***12,606***	***12,606***			
≤ 49 years	4724 (37.6)	183 (15.8)		4229 (36.6)	162 (15.6)					0.003
50–59 years	3782 (30.1)	282 (24.3)	1.92	3477 (30.1)	246 (23.6)	1.85	1.42	1.13	1.79	
60–69 years	3007 (23.9)	429 (37.0)	3.68	2851 (24.7)	390 (37.5)	3.57	1.60	1.22	2.10	
≥ 70 years	1059 (8.4)	267 (23.0)	6.51	1008 (8.7)	243 (23.3)	6.29	1.64	1.14	2.35	
Gender, *n* (%)										
*N*	***12,572***	***1161***	***13,733***	***11,565***	***1041***	***12,606***	***12,606***			
Female	6733 (53.6)	540 (46.5)		6228 (53.9)	493 (47.4)					0.020
Male	5839 (46.4)	621 (53.5)	1.33	5337 (46.1)	548 (52.6)	1.30	0.85	0.74	0.97	
National Index of Multiple Deprivation, *n* (%)										
*N*	***12,547***	***1157***	***13,704***	***11,565***	***1041***	***12,606***	***12,606***			
Quintile 1 (least deprived)	1731 (13.8)	174 (15.0)		1685 (14.6)	165 (15.9)					0.501
Quintile 2	2817 (22.5)	248 (21.4)	0.88	2711 (23.4)	232 (22.3)	0.87	0.88	0.71	1.10	
Quintile 3	1962 (15.6)	173 (15.0)	0.88	1856 (16.0)	161 (15.5)	0.89	0.88	0.67	1.14	
Quintile 4	3329 (26.5)	315 (27.2)	0.94	3029 (26.2)	271 (26.0)	0.91	0.96	0.75	1.23	
Quintile 5 (most deprived)	2708 (21.6)	247 (21.3)	0.91	2284 (19.7)	212 (20.4)	0.95	1.06	0.82	1.38	
Ethnicity, *n* (%)										
*N*	***12,572***	***1161***	***13,733***	***11,565***	***1041***	***12,606***	***12,606***			
White ethnicities	10,599 (84.3)	1013 (87.3)		9997 (86.4)	932 (89.5)					0.751
Mixed	365 (2.9)	18 (1.6)	0.52	289 (2.5)	15 (1.4)	0.56	0.96	0.49	1.87	
Asian or British Asian	478 (3.8)	46 (4.0)	1.01	373 (3.2)	28 (2.7)	0.81	0.97	0.65	1.45	
Other Black	485 (3.9)	29 (2.5)	0.63	349 (3.0)	22 (2.1)	0.68	1.16	0.76	1.77	
Other ethnic groups	207 (1.6)	17 (1.5)	0.86	190 (1.6)	14 (1.3)	0.79	1.31	0.82	2.08	
Not stated	438 (3.5)	38 (3.3)	0.91	367 (3.2)	30 (2.9)	0.88	0.84	0.62	1.15	
QRisk, *n* (%)										
*N*	***11,583***	***1043***	***12,626***	***11,565***	***1041***	***12,606***	***12,606***			
Low	7447 (64.3)	329 (31.5)		7435 (64.3)	329 (31.6)					< 0.001
Medium	2950 (25.5)	323 (31.0)	2.48	2947 (25.5)	323 (31.0)	2.48	2.04	1.65	2.52	
High	1186 (10.2)	391 (37.5)	7.46	1183 (10.2)	389 (37.4)	7.43	6.16	4.51	8.40	

Notes

* Prescribed /not prescribed a cardiovascular drug within three months of completing and NHS Health Check

OR presented for being prescribed a cardiovascular drug after controlling for age, gender IMD, ethnicity and Q-risk score

a. Odds ratios reported from logistic regressions clustered by practice (33 clusters), dependent variable: prescribed CVD drug within three months of attending NHS Health Check

b. Adjusted odds ratios reported from logistic regression clustered by practice (33 clusters) for model with age, gender, IMD, ethnicity and QRisk; dependent variable: prescribed CVD drug within three months of attending NHS Health Check

c. 95% confidence intervals reported for adjusted odds ratios (model with age, gender, IMD and ethnicity)

d. p-values reported for Wald tests in model with age, gender, IMD and ethnicity

e. The NHS Health Check program is not intended for under 40s, however a small number of patients were reported as < 40

Bold data represent absolute totals.

After controlling for age, gender, IMD quintile, ethnicity and QRisk score, compared to men, women were most likely to be prescribed a cardiovascular drug, (OR 1.18, 95% CI 1.03 to 1.35) as were patients aged ≥ 70 years compared to aged ≤ 70 years (OR 1.64, 95% CI 1.14 to 2.35). Those classified as being at high risk of CVD were most likely to be prescribed cardiovascular medication (OR 6.16, 95% CI 4.51 to 8.40). There was no evidence of any association between prescribing of CVD drugs and socioeconomic status or ethnicity (Table 3).

*Referral to behavioural lifestyle services*: A total of 695 recorded referrals were made to behavioural lifestyle services (Table 4) within eight-weeks following completion of an NHS Health Check: Weight management *n* = 414, Smoking cessation service *n* = 250, Physical activity service *n* = 37, Dietician *n* = 26, Health trainer *n* = 13, Alcohol service *n* = 1. Additionally, a further 10,381 patients were either signposted or given lifestyle advice within eight weeks following their NHS Health Check.

**Table 4:** Characteristics of patients referred (Yes) not referred (No)to lifestyle service*

Referred to lifestyle services within eight weeks following NHS Health Checks	Maximum N (*n* = 13,725)			Complete cases (*n* = 12,606)						
	No	Yes	Crude OR ^a^	No	Yes	Crude OR ^a^	Adjusted OR ^b^	95% CI ^c^		p-value ^d^
Age ^e^, *n* (%)										
*N*	***13,038***	***687***	***13,733***	***11,973***	***633***	***12,606***				
≤ 49 years	4608 (35.3)	299 (43.0)		4118 (34.4)	273 (43.1)					< 0.001
50–59 years	3829 (29.4)	235 (33.8)	0.95	3508 (29.3)	215 (34.0)	0.92	0.79	0.61	1.02	
60–69 years	3318 (25.4)	118 (17.0)	0.55	3137 (26.2)	104 (16.4)	0.50	0.33	0.25	0.44	
≥ 70 years	1283 (9.8)	43 (6.2)	0.52	1210 (10.1)	41 (6.5)	0.51	0.24	0.16	0.36	
Gender, * n *(%)										
*N*	***13,038***	***695***	***13,733***	***11,973***	***633***	***12,606***				
Female	6814 (52.3)	459 (66.0)		6303 (52.6)	418 (66.0)					< 0.001
Male	6224 (47.7)	236 (34.0)	0.56	5670 (47.4)	215 (34.0)	0.57	0.45	0.34	0.59	
National Index of Multiple Deprivation, * n* (%)										
*N*	***13,017***	***687***	***13,704***	***11,973***	***633***	***12,606***				
Quintile 1 (least deprived)	1857 (14.3)	48 (7.0)		1803 (15.1)	47 (7.4)					0.015
Quintile 2	2949 (22.7)	116 (16.9)	1.52	2832 (23.7)	111 (17.5)	1.50	1.45	1.07	1.97	
Quintile 3	2043 (15.7)	92 (13.4)	1.74	1928 (16.1)	89 (14.1)	1.77	1.67	0.99	2.79	
Quintile 4	3453 (26.5)	191 (27.8)	2.14	3130 (26.1)	170 (26.9)	2.08	1.87	1.13	3.12	
Quintile 5 (most deprived)	2715 (20.9)	240 (34.9)	3.42	2280 (19.0)	216 (34.1)	3.63	3.22	1.63	6.36	
Ethnicity, *n* (%)										
*N*	***13,038***	***695***	***13,733***	***11,973***	***633***	***12,606***				
White	11,008 (84.4)	604 (86.9)		10,368 (86.6)	561 (88.6)					0.078
Mixed	359 (2.8)	24 (3.5)	1.22	283 (2.4)	21 (3.3)	1.37	1.09	0.63	1.88	
Asian or British Asian	502 (3.9)	22 (3.2)	0.80	387 (3.2)	14 (2.2)	0.67	0.47	0.19	1.14	
Other Black	484 (3.7)	30 (4.3)	1.13	347 (2.9)	24 (3.8)	1.28	0.91	0.44	1.87	
Other ethnic groups	216 (1.7)	8 (1.2)	0.68	197 (1.6)	7 (1.1)	0.66	0.58	0.23	1.49	
Not stated	469 (3.6)	7 (1.0)	0.27	391 (3.3)	6 (0.9)	0.28	0.31	0.12	0.76	
QRisk, * n* (%)										
*N*	***11,989***	***637***	***12,626***	***11,973***	***633***	***12,606***				
Low	7353 (61.3)	423 (66.4)		7342(61.3)	422 (66.7)					< 0.001
Medium	3131 (26.1)	142 (22.3)	0.79	3129 (26.1)	141 (22.3)	0.78	1.68	1.38	2.05	
High	1505 (12.6)	72 (11.3)	0.83	1502 (12.5)	70 (11.1)	0.81	2.77	1.91	4.02	

Notes

*Referred (Yes), or not referred (No, to a lifestyle service within three months following an NHS Heath Check

OR presented for being referred to a lifestyle service after controlling for age, gender IMD, ethnicity and Q-risk score.

^a^ Odds ratios reported from logistic regressions clustered by practice (37 clusters), dependent variable: referred to lifestyle service within three months of attending NHS Health Check

^b^ Adjusted odds ratios reported from logistic regression clustered by practice (37 clusters) for model with age, gender, IMD, ethnicity and QRisk; dependent variable: referred to lifestyle service within three months of attending NHS Health Check

^c^ 95% confidence intervals reported for adjusted odds ratios (model with age, gender, IMD and ethnicity)

^d^ *p*-values reported for Wald tests in model with age, gender, IMD and ethnicity

e. The NHS Health Check program is not intended for under 40s, however a small number of patients were reported as < 40

Bold data represent absolute totals.

The following groups were most likely to be referred to lifestyle services: younger women (OR 2.22, 95% CI 1.69 to 2.94), those in the most deprived IMD quintile (OR 3.22, 95% CI 1.63 to 6.36) and those who were at highest risk of CVD (OR, 2.77, 95% CI 1.91 to 4.02).

## Discussion

### Summary

Over the data collection period, 31,881 invitations for an NHS Health Check were offered to eligible individuals and 13,733 Checks were completed. Although 52 GP practices were offered the opportunity to deliver NHS Health Checks and or take part in this study, only 32 practices were able to arrange the infrastructure required to do this, in the time frame for this study. Analysis of all practices who participated as well as those who didn’t, showed that IMD was normally distributed. Slightly more males than females were invited (52% versus 48%) although slightly fewer males than females attended (47% versus 53%). More patients, classified as white British, mixed British, other white and white European [17], compared to those from other BAME groups were invited (64% versus 9%). From those who were invited, the majority of attendees were also from white British, mixed British, other white and white European [17], compared to those from all other BAME groups (85% versus 10%). The population prevalence of BAME groups in the City of Bristol is 16%.

There was no statistically significant difference across IMD quintiles in invitations made or attendance for an NHS Health Check. However, for attendance, there was a trend for those in the most socially and economically deprived IMD quintiles, to be least likely to attend for their NHS Health Check, compared to patients from least socially and economically deprived IMD quintiles (*P* = 0.053); 95% confidence intervals included one for all IMD quintiles. A higher percentage of those invited and who completed their NHS Health Check had a low Qrisk score (61.6%) compared to those with a medium (26.0%) or high-risk score (12.5%). Age and gender were significant predictors of attendance with older patients and females, most likely to attend.

From the patients who were invited and attended, compared to those with a low Qrisk score, those with a high QRisk score were more likely to be prescribed a cardiovascular drug and referred to a behavioural lifestyle management service. Those referred to behavioural lifestyle management were more likely to be living in the most socially and economically deprived areas compared to living in the least socially and economically deprived areas.

### Comparison with existing literature

Early identification of risk factors for CVD is important to more accurately inform primary prevention. It is currently unclear whether the NHS Health Checks programme is able to increase the early identification of CVD risk factors.

It is difficult to ascertain whether NHS Health Checks attract patients at highest risk of CVD, as recommendations in some areas have been to invite higher risk patients first [19]. Whilst others have found non-attenders to have a higher set of risk factors for CVD compared to attenders [20], several studies, including ours have found a greater rate of attendance in older patients [21], a population more likely to have a higher set of risk factors for CVD.

However, in our study, within three months of attending for an NHS Health Check, just over 8% of patients, of which over a third (almost 38%) had high QRisk scores, were prescribed a cardiovascular drug; with a high QRisk score being a significant predicator for being prescribed a CVD drug. Thus, providing some support that those who attended and were at highest risk of CVD, were being identified and treated.

Additionally, just over 5% of those who attended were referred to lifestyle services, with younger women and those from the most socially and economically deprived areas in Bristol most likely to be referred. These are patients who if they hadn’t been invited and attended for an NHS Health Check, would not have been identified as being at risk of CVD until much later, when their risk factors may have advanced or resulted in a cardiovascular event. This provides some positive support for the NHS Health Checks programme in this geographical location. A randomised trial of NHS Health Checks with and without referral to behavioural lifestyle support showed a reduction in estimated population CVD risk, with CVD risk being assessed using the Framingham 10-year CVD risk guidelines [22]. There was no difference in risk reduction between groups, indicating that the addition of behavioural lifestyle support made no significant difference in this case. However, in our study, we did not have data available to examine the impact of referral, to behavioural lifestyle support on risk reduction in our population.

More recent evidence found an increase in the new diagnosis of particularly diabetes and hypertension among attendees compared to non-attendees of an NHS Health Check, as well as a higher level of statin prescribing [19, 23]. These findings further contribute towards positive support for the NHS Health Checks programme.

In contrast, a recent study examining the impact of NHS Health Checks on the prevalence of risk factors for CVD [24] found no difference in prevalence, between practices who did or did not offer NHS Health Checks. This was despite the practices offering NHS Health Checks being in more deprived and low-income communities where there is likely to be a greater prevalence of risk factors for CVD, compared to the control practices [25].

Inequalities in health arise from social, geographical or biological differences between people or groups of people. Often classified as ‘deprived or disadvantaged groups’, they live in the poorest neighbourhoods, are on low incomes and often removed from access to basic needs such as food, a safe living environment, health and social services. These conditions tend to result in poorer population health compared to those living in less deprived neighbourhoods [26] .This population often includes a high proportion of those from BAME groups. According to the Marmot curve [27], a social gradient in health exists, where populations with higher levels of income deprivation have lower life expectancy and less disability-free years. In the UK, mortality from CVD is 50% higher in those who are in the most deprived fifth of the population compared to those in the most affluent fifth of the population [6]. Uptake of NHS Health Checks based on patient levels of deprivation and or income, has been equivocal. Whilst some studies have reported that NHS Health Checks attract those at higher risk of CVD, including those from low income groups [21], others have reported a reduced uptake amongst those from low compared to higher income groups [28] or even no differences across socioeconomic groups [11, 12]; we found the latter in our study.

Indeed, many programmes aimed at either screening or the early identification of disease, tend to be accessed predominantly by more affluent, healthier individuals [5, 20]. This raises the question ‘how effective are NHS Health Checks in reaching those populations who live in low income communities and who are at highest risk of CVD?’ Our study didn’t indicate that being in the least affluent sector of the population was a significant predictor for attendance. However, there were a higher proportion of invites sent and patients who attended for their NHS health Check, from the least compared to the most deprived quintile. This may merely be a reflection of targeting by the practices in this study, but it wasn’t possible to ascribe a uniform stratification pattern for how practices invited their population.

Several factors can lower rates attendance for an NHS Health Check in those living in low income communities. This may include patient’s scepticism about the efficacy of an NHS Health Check or valuing health less strongly than attenders. Some studies have used a theoretical model of health behaviour change [29], to explain non-attendance [20, 30–32], whilst the inverse care law also goes someway to explaining this likelihood [33]. In a study of community outreach clinics being offered in a deprived area of Bristol, some patients voiced that by offering the NHS Health Check at a community outreach clinic, it showed a willingness by the health care professionals to get to know the patients because they are ‘coming out to see us’ at a community venue, making the patient feel valued and heard [34]. However, it is likely that the explanation is complex with many different factors impacting on the decision to attend. Examples may include: patients having moved or re-located and not left a forwarding address, language difficulties or lack of an interpreter as well as the location and timing of the appointment [34].

The prevalence of CVD and diabetes in many ethnic minority populations, especially those of south Asian origin is higher than that found in Caucasian populations [35, 36]. A recent study conducted in Luton, UK, an ethnically diverse town, found that patients from ‘other white backgrounds’ and ‘Black African’ patients, were less likely to attend for their NHS Health check compared to all other ethnic groups [27, 28]. This finding also concurs with that found in other studies [20, 37, 38] as well as our own, where only 10% of attendees for an NHS Health check were from BAME populations, compared to a population prevalence in Bristol of 16%. Where targeted approaches for inviting BAME groups have been used, this has been more effective in encouraging BAME patients to attend, compared with an un-targeted, general population approach [10, 34, 39].

Previous studies have reported a lower uptake of NHS Health Checks amongst younger eligible patients [10, 11, 21, 28]. This concurs with our own findings, where being aged ≥ 50 was a significant predictor for attendance, with increasing age improving the likelihood of attendance. Work and or family commitment in those under the age of 50 years, may have contributed towards this lower rate of attendance.

Several studies, including ours, have highlighted gender as a predictor of attendance for an NHS Health Check [37, 38], with women being more likely than men to attend. Women, more often, attend their GP practice, compared to men [25], increasing the likelihood for an opportunistic NHS Health Check.

Previous studies have shown that the method of inviting patients for an NHS Health Check is a predictor for attendance [28, 37, 38], with verbal, telephone, and enhanced letter invitations being predictors of attendance, compared to a traditional letter invite.

Additionally, targeted approaches to prioritise and invite patients with the highest estimated risk for CVD, for an NHS Health Check have been more effective [40] and cost effective [41] at encouraging attendance, compared to a general population approach. These studies selected patients using case finding, informed by estimating each patient’s CVD risk, calculated from routinely collected general practice data. Compared with a control period, targeting high-risk cases increased the number of patients requiring antihypertensive and or statin treatment.

In summary, findings are equivocal on the equity of uptake for NHS Health Checks. Some of these differences may be due to differing geographical settings of studies and or methodological differences between the studies.

### Limitations of this study

Some practices prioritised inviting patients who were at higher risk of CVD, in addition to offering NHS Health Checks opportunistically. However, there was no consistency in prioritising invites across or between the practices included in this study. This makes it difficult to fully assess the equity of invitations made and uptake of NHS Health Checks among the included practices.

In 2010, when data collection for this study started, the NHS Heath Checks programme was a new initiative to GP practices in Bristol. GP practices included in this study increased their rate of completed NHS Health Checks per month, over the data collection period, from 68.4 per month in 2010, to 459.8 per month. This indicates that practices became more focused in their efforts over this time.

Ethnicity is a key component for calculating the QRisk score. Ethnicity was routinely, poorly collected and impacted on our ability to assess equity of uptake, beyond a descriptive analysis. This was particularly the case for patients who were invited but did not attend for their NHS Health Check, where 45% of data on ethnicity was missing. This limitation is consistent with other similar studies [28].

Referral to behavioural lifestyle services was poorly recorded at practice level, according to reports from the GP practices and commissioners of NHS Health Checks associated with our study. These practices reported that patients did not always accept the offer of a referral to behavioural or lifestyle services directly after their NHS Health Check. They often made the decision at a later date. This subsequent referral was not always captured in the patient’s electronic medical record.

## Conclusions

In conclusion, although we observed a trend in inequity of attendance among those in lower social-economic groups, this did not reach statistical significance. However, being ≥ 50 and female, were significant predictors of attendance. Having a high QRisk score, being female and being aged ≤ 49 were predictors of being prescribed CVD medications.

Bristol has a diverse ethnic population, where 16% are from BAME populations and there exists diffuse areas of deprivation. This study provides a local perspective on the uptake of NHS Heath Checks. It identifies gaps in equity of uptake, where provision across eligible populations is less effective. Our findings support the need for local commissioners, primary care and related lifestyle service providers to better target approaches at men and younger populations, as well as BAME populations, where uptake doesn’t match population prevalence. As a result of the trend in inequality of uptake, among socially and economically deprived communities, targeted approaches should be further explored and developed.

Additionally, it contributes to the evidence base for the current efficacy of NHS Health Checks, particularly in women and older eligible populations, where the reach in provision is effective.

Our study points to the importance of targeted approaches for younger patients and men, whose annual mortality from CVD under the age of 74, is higher than that in women [1]. Offering appointments at varied times may help to address the needs of eligible, working patients. Targeted approaches at men could include offering NHS Health Checks in male dominated workplaces or social environments. Attendance among BAME groups was poor and targeted approaches to address this should be developed. General practices should be encouraged to routinely record ethnicity. This will help contribute to the early identification of CVD risk in patients from BAME groups; this is important due to the higher risk of CVD in some BAME populations compared to non-BAME populations. In view of the trend for lower attendance for an NHS Health Check among patients from more deprived IMD quintiles, strategies to encourage attendance in this group of patients should be implemented. The management of comorbidities and CVD risk as well as more accurate recording of referrals to behavioural or lifestyle services, and long-term follow-up of the outcomes from this, need further exploration. This will identify whether available services are being used to facilitate patients in the self-management of modifiable risk factors identified during their NHS Health Check. It will also encourage a better engagement with these services if necessary. These aspects are fundamental if we are to maximise the impact of early identification of modifiable risk factors resulting from NHS Health Checks. This will assist not only with NHS Health Checks, but other related, primary care activities.

## Abbreviations

BAME: Black and Asian minority ethnic groups
CLAHRC: Collaboration for leadership in applied health research and care west
CSU: Commissioning support unit
CVD: Cardiovascular disease
IMD: Index of multiple deprivation
NIHR: National institute of health research
SES: Socio economic status
SPCR: School of primary care research

## References

[1] British Heart Foundation (2015). Cardiovascular Disease Statistics 2015.

[2] British Heart Foundation. 2014. https://www.bhf.org.uk/news-from-the-bhf/news-archive/2014/august/rising-cost-of-cvd . Accessed 22 Nov 2016.

[3] Lim SS, et al. A comparative risk assessment of burden of disease and injury attributable to 67 risk factors and risk factor clusters in 21 regions, 1990-2010: a systematic analysis for the global burden of disease study 2010. Lancet. 2012;380 10.1016/S0140-6736(12)61766-8 PMC4156511 23245609

[4] NHS Choices. http://www.nhs.uk/Conditions/nhs-health-check/Pages/NHS-Health-Check.aspx. Accessed June 2017.

[5] McCartney M (2012). The Patient Paradox. Why Sexed Up Medicine is Bad for Your Health.

[6] Heart UK (2013). The cholesterol charity. Bridging the gap.

[7] British Heart Foundation Ethnic differences in coronary heart disease. British heart foundation statistics database 2010. www.heartstats.org. Accessed Mar 2017.

[8] Public Health England Publications (2015). Health inequalities briefing for London NHS Health Checks and target diseases: Inequalities by protected characteristics and socioeconomic factors.

[9] Department of Health. Living Well for Longer: A Call to Action to Reduce Avoidable Premature Mortality: Public Health Policy & Strategy Unit/NHS Commissioning Unit. London: DOH, 2013.

[10] Roberts DJ, de Souza VC. A venue-based analysis of the reach of a targeted outreach service to deliver opportunistic community NHS health checks to ‘hard to reach’ groups. Public Health. 2016;137 10.1016/j.puhe.2016.03.004 27062066

[11] Chang KC (2015). Coverage of a cardiovascular risk assessment and managements programme (NHS health check): retrospective database study. Prev Med.

[12] Robson J, Dostal I, Madurasinghe V, et al. The NHS health check programme: implementation in East London. BMJ Open. 2015; 10.1136/bmjopen-2015-007578. Accesed September 25. 10.1136/bmjopen-2015-007578 PMC4401839 25869692

[13] Bristol City Council. The population of Bristol. https://www.bristol.gov.uk/documents/20182/33904/Population+of+Bristol+July+2016/858ff3e1-a9ca-4632-9f53-c49b8c697c8c. (1). Accesed July 2017.

[14] Department for Communities and Local Government The English indices of deprivation. Neighbourhood statistics release. 2015. https://www.gov.uk/government/collections/english-indices-of-deprivation. Accessed 4 May 2017.

[15] Office for National Statistics Census. Count me in Census England Household Form. 2017. https://www.ons.gov.uk/peoplepopulationandcommunity/culturalidentity/ethnicity/articles/ethnicityandnationalidentityinenglandandwales/2012-12-11#ethnicity-in-englandand-wales. Accessed 4 May 2017.

[16] Hippisley-Cox J, Coupland C, Robson J, Brindle P. Derivation, validation, and evaluation of a new QRISK model to estimate lifetime risk of cardiovascular disease: cohort study using QResearch database. BMJ. 2010;9, 341 10.1136/bmj.c6624 PMC2999889 21148212

[17] Ethnicity and first language recording - GPC guidance, 2012. https://www.bma.org.uk/advice/employment/contracts/gp-partner-contracts/desethnicity. Accessed Nov 2015.

[18] Von Elm E, Altman DG, Egger M, Pocock SJ, Gøtzsche PC, Vandenbroucke JP, STROBE Initiative (2008). The strengthening the reporting of observational studies in epidemiology (STROBE)statement: guidelines for reporting observational studies. J Clin Epidemiol.

[19] Robson J, Dostal I, Madurasinghe V, Sheikh A, Hull S, Boomia K, Griffiths C, Eldridge S (2017). NHS health check comorbidity and management: an observational matched study in primary care. Br J general practice.

[20] Dryden R, Williams B, McCowan C, Themessl-Huber M (2012). What do we know about who does and does not attend general health checks? Findings from a narrative scoping review. BMC Public Health.

[21] Robson J, Dostal I, Sheikh A, et al. The NHS health checks in England: an evaluation of the first 4 years. BMJ Open. 2016; 10.1136/%20bmjopen-2015-008840. 10.1136/bmjopen-2015-008840 PMC4735215 26762161

[22] Cochrane T, Davey R, Iqbal A, Gidlow C, Kumar J (2012). NHS Health checks through general practice: randomised trial of population cardiovascular risk. BMC Public Health.

[23] Chang KCM, Lee JT, Vamos EP, Soljak M, Johnston D, Khunti K, Majeed A, Millett C. Impact of the National Health Service health check on cardiovascular disease risk: a difference-in-differences matched analysis. Canadian Med Association. 2016;188(10) 10.1503/cmaj.151201 PMC4938708 27141033

[24] Caley M, CP HJ, Wright N (2014). The impact of NHS health checks on the prevalence of disease in general GP practices: a controlled study. Br J Gen Pract.

[25] Lang SJ, Abel GA, Mant J, Mullis R. Impact of socioeconomic deprivation on screening for cardiovascular disease risk in a primary prevention population: a cross-sectional study. BMJ Open. 2016; 10.1136/bmjopen-2015-009984. 10.1136/bmjopen-2015-009984 PMC4809080 27000783

[26] NICE. Health inequalities and population health. https://www.nice.org.uk/advice/lgb4/chapter/introduction (2012). Accessed 4 May 2017.

[27] The Marmot review). Fair society healthy lives. The Marmot Review. Strategic view of health inequalities in England post 2010. http://www.instituteofhealthequity.org/resources-reports/fair-society-healthy-lives-the-marmot-review. Accessed 4 May 2017.

[28] Cook EJ, Sharp C, Randhawa G, Guppy A, Cox J (2016). Who uses NHS health checks? Investigating the impact of ethnicity and gender and method of invitation on uptake of NHS health check. BMC Int J Equity Health.

[29] Becker MH (1974). The health belief model and personal health behaviour.

[30] Simpson WM, Johnson M, McEwan SR (1993). Screening for risk factors or cardiovascular disease: a psychological perspective. Scott Med J.

[31] Hsu HY, Gallinagh R. The relationship between health beliefs and utilization of free health examinations in older people living in a community setting in Taiwan. J Advanced Nursing. 2001;35(6) 10.1046/j.1365-2648.2001.01924.x 11555034

[32] Shiph S, Vniter M, Barak Z (1997). Correlates of health screening utilization: the roles of health belief and self-motivation. Psychol Health.

[33] Hart JT (1971). The inverse care law. Lancet.

[34] Riley R, Coghill N, Montgomery A, Feder G, Horwood J (2015). The provision of NHS health checks in a community setting: an ethnographic account. BMC Health Service Research.

[35] Solutions for public health (2011). Obesity and ethnicity. National obesity observatory Oxford.

[36] British Heart Foundation Health Promotion Group. Ethnic differences in cardiovascular disease. File:///C:/users/Nikki/documents/NHS%20health%20checks/writing%20up/BMC/hs2010fc_ethnic_differences_in_cardiovascular_disease-full-copy.Pdf. (2010). Accessed 4 May 2017.

[37] Gidlow C, Ellis N, Randall J, et al. Methods of invitation and geographical proximity as predictors of NHS health check uptake. J Public Health (Oxford). 2014;(2) 10.1093/pubmed/fdu092 PMC4447133 25427882

[38] Sallis A, Bunton A, Bonus A, James A, Chadborn T, Berry S. The effectiveness of an enhanced invitation letter on uptake of National Health Service Health Checks in primary care: a pragmatic quasi-randomised controlled trial. BMC Fam Pract. 2016;7(35) 10.1186/s12875-016-0426-y PMC4806508 27009045

[39] Hunt K, Adamson J, Hewitt C, Nazareth I. Do women consult more than men? A review of gender and consultation for back pain and headache. J Health Serv Res Policy. 2011;16(2) 10.1258/jhsrp.2010.009131 PMC3104816 20819913

[40] Hemming K, Ryan R, Gill P, Westerby P, Jolly K, Marshall T. Targeted case finding in the prevention of cardiovascular disease: a stepped wedge cluster randomised controlled trial. Br J Gen Pract. 2016;66(651) 10.3399/bjgp16X686629 PMC5033312 27528707

[41] Crossan C, Lord J, Ryan R, Nherera L, Marshall T. Cost effectiveness of case-finding strategies for primary prevention of cardiovascular disease: a modelling study. Br J Gen Pract. 2017;67(654) 10.3399/bjgp16X687973 PMC5198616 27821671

